# Perforated Diverticulitis Presenting as Acute Leg Pain and Inability to Bear Weight: An Atypical Manifestation of a Common Pathology

**DOI:** 10.7759/cureus.84272

**Published:** 2025-05-17

**Authors:** Alizatu Koroma, Rachel A Daley, Alyssa McMandon, Saptarshi Biswas

**Affiliations:** 1 Surgery, Trident Health System, North Charleston, USA; 2 Medical Student, Edward Via College of Osteopathic Medicine, Spartanburg, USA; 3 Surgery, Grand Strand Medical Center, Myrtle Beach, USA

**Keywords:** fistula formation, necrotizing myositis, perforated diverticulitis, retroperitoneal abscess, thigh gas

## Abstract

Acute diverticulitis commonly presents with abdominal symptoms but can rarely lead to extra-abdominal complications such as lower extremity necrotizing myositis. While fistulas involving the bladder or vagina are well described, retroperitoneal spread with extension into the thigh remains exceedingly uncommon. We present the case of a 77-year-old female who arrived with acute left thigh pain, swelling, and inability to bear weight. Initial workup was negative for deep vein thrombosis, but further history revealed preceding abdominal and back pain. Computed tomography identified extraluminal gas tracking from a perforated sigmoid colon into the retroperitoneum and anterior thigh. Exploratory laparotomy confirmed perforated diverticulitis with retroperitoneal fistulization and abscess formation extending into the thigh musculature. Surgical management included Hartmann’s procedure, retroperitoneal washout, and debridement of necrotic thigh tissue. The patient recovered successfully following an intervention. This case underscores a rare but critical complication of diverticulitis, emphasizing the importance of considering intra-abdominal sources when encountering unexplained gas in the thigh. Timely diagnosis and aggressive surgical management are essential for optimal patient outcomes.

## Introduction

Diverticular disease is one of the most prevalent gastrointestinal conditions encountered in both inpatient and outpatient settings. Acute diverticulitis arises from microscopic or macroscopic perforation of the diverticulum, typically due to localized inflammation and focal necrosis [1]. It is estimated that 10-25% of individuals with diverticulosis will develop diverticulitis in their lifetime [1,2]. 

Clinical presentations range from mild, uncomplicated cases to severe, complicated forms associated with stricture formation, abscesses, bowel obstruction, perforation, and fistula development. Perforated diverticulitis, in particular, is a potentially life-threatening complication that may result in peritonitis, intra-abdominal abscesses, or systemic sepsis due to the translocation of fecal material and bacteria into the peritoneal cavity. In rare instances, the inflammatory and infectious process may extend beyond the peritoneum and involve retroperitoneal structures, leading to atypical extra-abdominal complications.

We describe a rare presentation of perforated diverticulitis with retroperitoneal extension resulting in a complex abscess that tracked along the iliopsoas compartment into the left thigh, ultimately causing necrotizing myositis. While thigh abscesses are most commonly attributed to localized soft tissue infections, trauma, or foreign body reactions, this case highlights the importance of considering intra-abdominal sources in the differential diagnosis of unexplained lower extremity infections.

This case report was presented as a Quickshot presentation at the Southeastern Surgical Congress 2023.

## Case presentation

A 77-year-old female presented to the emergency department with a three-day history of progressive left leg pain and swelling, resulting in difficulty bearing weight on the affected limb. The pain was localized primarily to the proximal medial thigh. She also reported a six-day history of sharp lower quadrant abdominal pain, which worsened with movement and radiated across the lower abdomen. Associated symptoms included one episode of non-bloody diarrhea and subjective fever. 

Her medical history was notable for previously treated diverticulitis, hypertension, gastroesophageal reflux disease (GERD), and dyslipidemia. She had undergone a colonoscopy five years prior but was unable to recall the findings. She denied the use of corticosteroids, immunosuppressants, or hormonal therapy and had no history of vasculitis or deep vein thrombosis (DVT).

**Figure 1:**
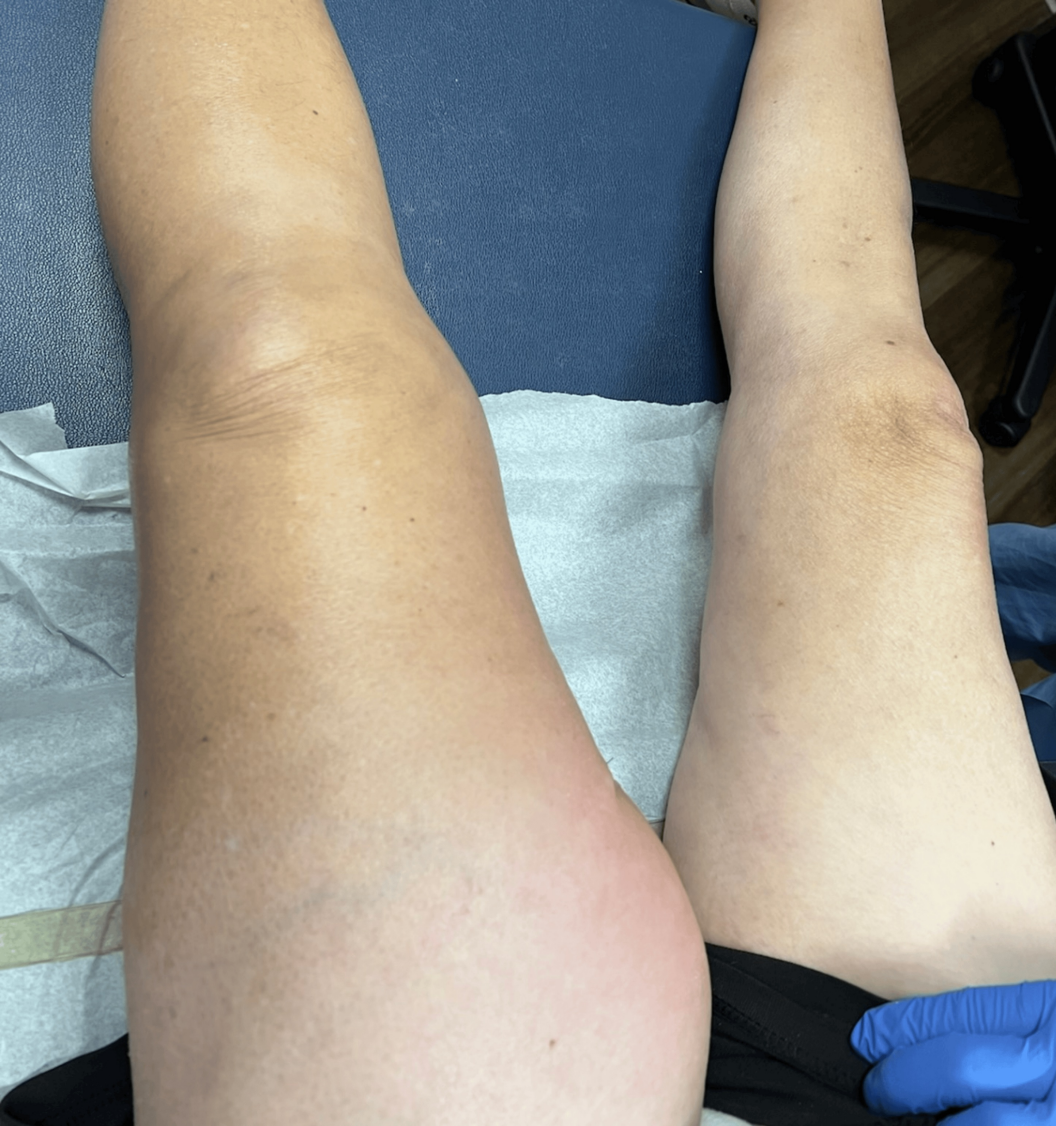
Early clinical presentation of left thigh involvement in perforated sigmoid diverticulitis with retroperitoneal extension

On presentation, her vital signs were: temperature 36.3 °C, blood pressure 110/59 mmHg, heart rate 89 bpm, and oxygen saturation 100% on room air. Physical examination revealed a doughy, mildly distended abdomen (Figure 1) with severe tenderness in the left lower quadrant, voluntary guarding, and rebound tenderness. An examination of the left proximal thigh showed tenderness and crepitus without overlying skin changes or discoloration. Laboratory findings were significant for mild leukocytosis (WBC 13.1, 91.3% neutrophils) and acute kidney injury (creatinine 1.95, BUN 22 mg/dL).

**Figure 2:**
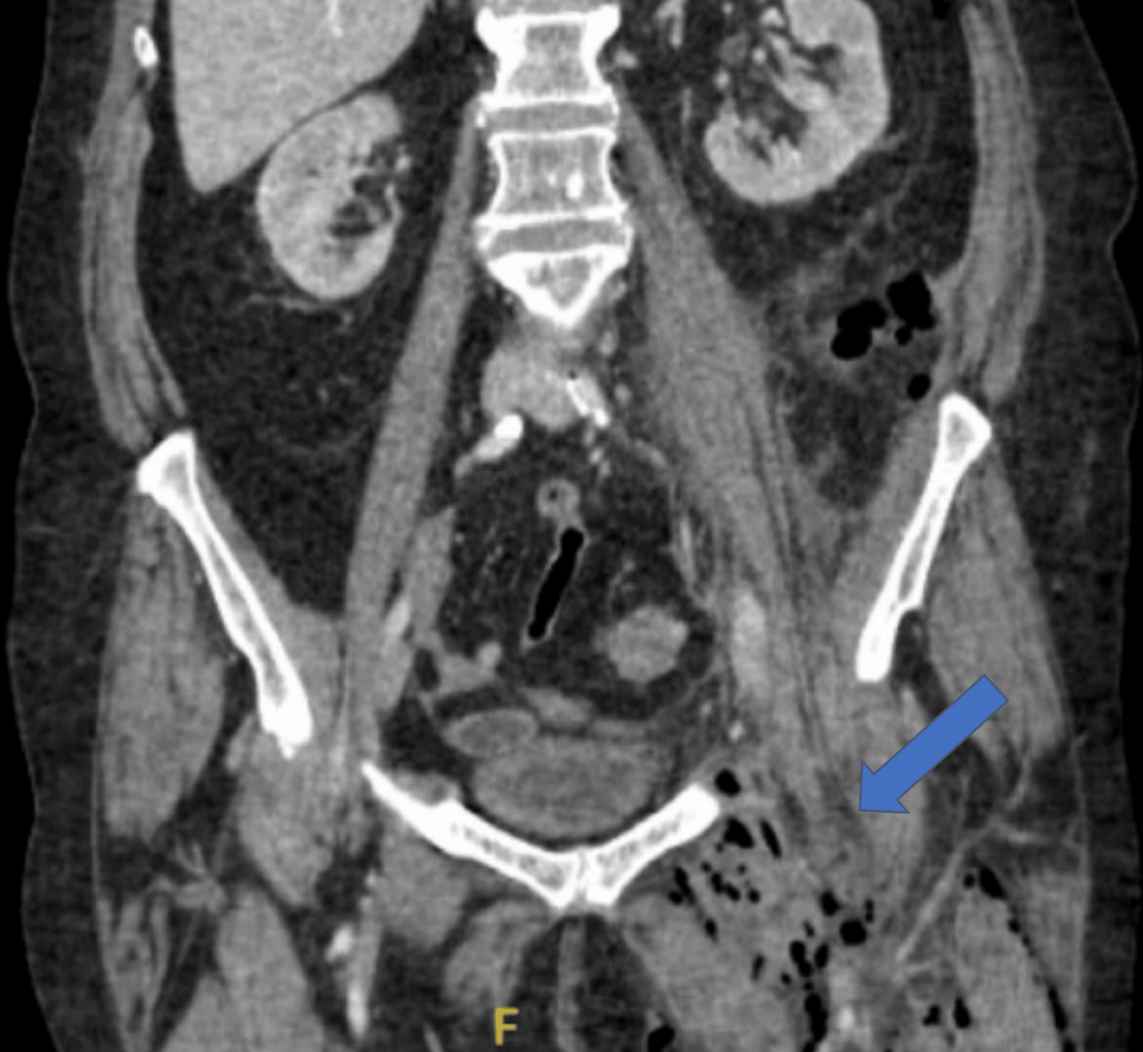
Presenting CT showing retroperitoneal gas extending into the proximal thigh along fascial planes The blue arrow highlights retroperitoneal gas.

**Figure 3:**
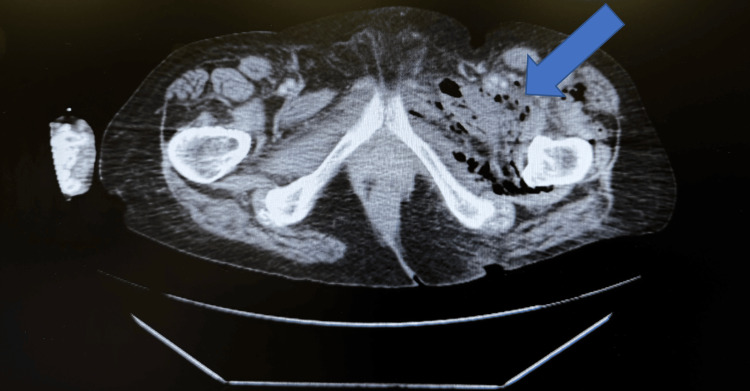
Axial CT showing extensive retroperitoneal gas extending into the pelvis, suggestive of soft tissue infection or gastrointestinal perforation with fascial plane tracking The blue arrow highlights retroperitoneal gas.

**Figure 4:**
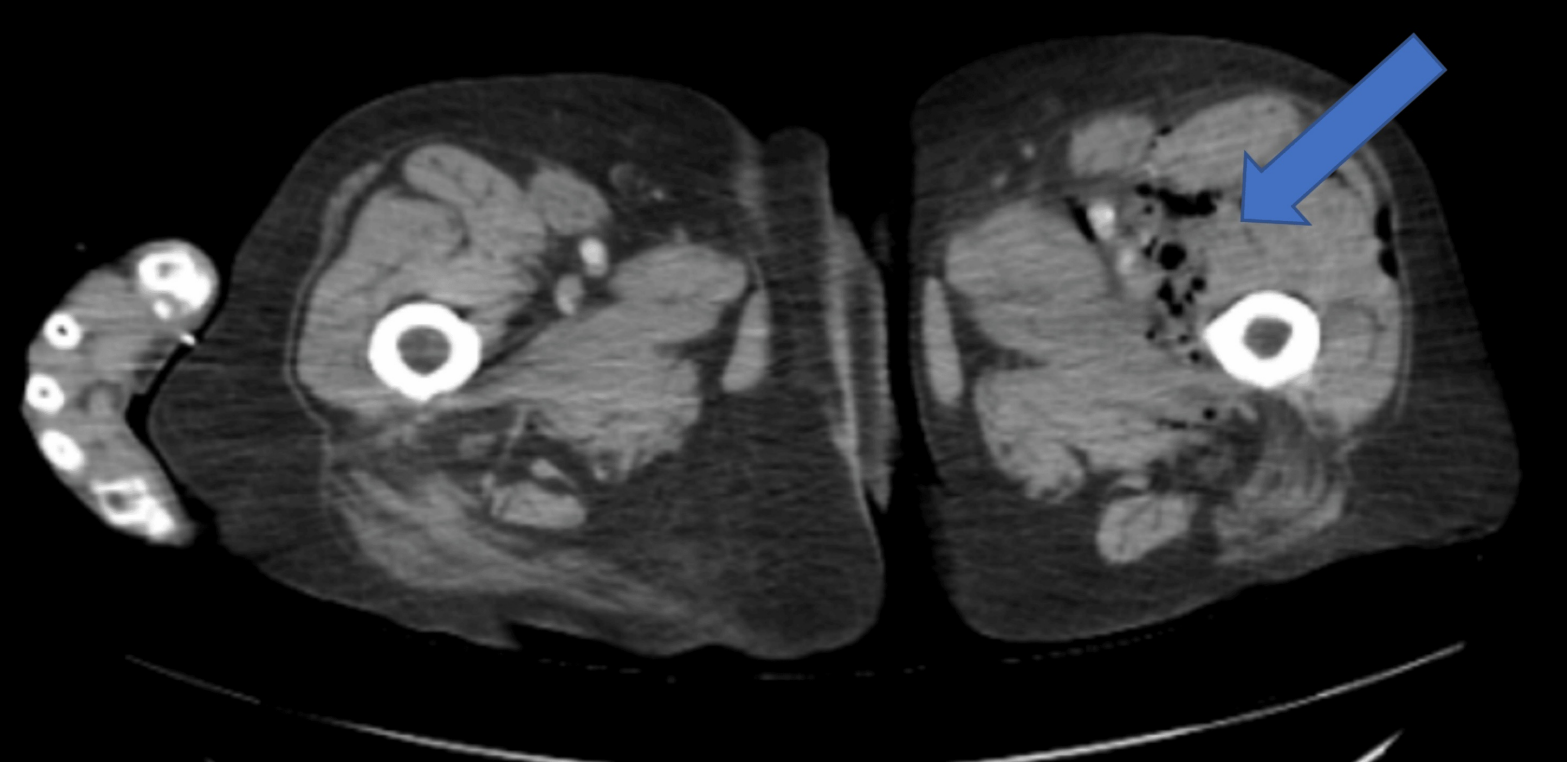
Axial CT demonstrating gas within the thigh musculature, consistent with extension of retroperitoneal infection and necrotizing soft tissue involvement The blue arrow highlights gas within the thigh musculature.

A bilateral lower extremity duplex ultrasound was negative for DVT. Computed tomography (CT) of the abdomen and pelvis revealed gas within the posterior paracolic gutter and left iliac fossa, extending into the soft tissues of the left thigh with evidence of intramuscular gas. The colon demonstrated diverticulosis and segmental wall thickening in the distal colon. The gas within the thigh was not directly contiguous with the colonic lumen, raising concern for a fistulous tract from a retroperitoneal source (Figures 2-4).

The patient was started on empiric broad-spectrum antibiotics, piperacillin-tazobactam, vancomycin, and clindamycin to provide coverage for intra-abdominal sepsis and suspected necrotizing soft tissue infection of the left lower extremity. Given the imaging findings and clinical presentation, she was taken emergently to the operating room for exploratory laparotomy. Intraoperatively, the sigmoid colon was found to be thickened, indurated, erythematous, and adherent to the retroperitoneum. Resection of the sigmoid colon revealed a retroperitoneal multiloculated abscess extending along the psoas muscle and perinephric space. The collection was drained, and the retroperitoneum was copiously irrigated. The patient was left in discontinuity with the ABThera™ vacuum-assisted closure (VAC) device (3M™, Saint Paul, Minnesota, US) in place and transferred to the intensive care unit (ICU) for further resuscitation.

The following day, she returned to the operating room for re-exploration. A Hartmann’s procedure was performed, and the abdominal fascia and skin were closed. Concurrently, exploration of the left thigh was performed, which revealed mostly viable muscle and subcutaneous tissue, though areas of nonviable tissue were identified and excised during surgical debridement. A surgical drain was placed. The patient was subsequently extubated and returned to the ICU. She underwent formal closure of the thigh incision the following day.

Her subsequent postoperative course was uncomplicated. She made a full recovery and was discharged to an acute rehabilitation facility for continued recovery and physical therapy. At monthly follow-up visits, she has remained clinically stable and continues to do well.

## Discussion

Thigh abscesses are typically caused by soft tissue infections, trauma, or foreign body reactions. However, in rare instances, they can be the result of an underlying intra-abdominal pathology. This case presents the rare complication of a lower extremity necrotizing myositis secondary to perforated acute diverticulitis.

Perforated diverticulitis, in some cases, can extend beyond the peritoneum and affect adjacent structures, leading to extraperitoneal complications. A well-known complication of perforated diverticulitis is a fistula such as a colovaginal fistula and colovesical fistula [3]. The inflammation from the perforation can form a fistula track along the mesocolon and retroperitoneum, resulting in extraperitoneal abscess collection. Thigh abscess as a result of a fistula of the retroperitoneum has been reported from perforation of a malignant colonic tumor [4-7].

There are few reports highlighting the routes for intra-abdominal infections to reach the thigh through either the femoral sheath, femoral canal, psoas sheath, sciatic notch, or obturator foramen [8-10]. For our patient, her thigh abscess was possibly through the psoas sheath because the abscess was seen in the retroperitoneum area around her psoas muscle and perinephric space. We performed Hartmann’s procedure with drainage of the retroperitoneal abscess and local external drainage of the thigh abscess.

Gas in the thigh should increase the index of suspicion of bowel pathology, especially with the absence of an infectious source. The main abdominal sources to consider in thigh abscesses are colorectal carcinoma, diverticulitis, ischiorectal abscess, appendicitis, Crohn’s disease, and tuberculous psoas abscess. Rapid recognition of the source of sepsis and adequate control with emergent exploration and debridement offer the best chance of a successful outcome.

## Conclusions

This case represents an exceedingly rare complication of perforated diverticulitis presenting as necrotizing myositis of the thigh, a manifestation scarcely documented in the literature. Unlike more commonly described fistulous extensions to the bladder or vagina, this case involved a retroperitoneal abscess tracking along the psoas sheath into the anterior thigh musculature. The absence of overt abdominal signs and initial imaging mimicking vascular pathology highlights the diagnostic challenges such cases pose. Our report underscores the importance of considering intra-abdominal etiologies in the differential diagnosis of unexplained soft tissue gas in the extremities. Early recognition, multidisciplinary coordination, and prompt surgical management were pivotal to the patient’s favorable outcome. This case expands the clinical spectrum of diverticulitis complications and reinforces the need for vigilance when evaluating atypical presentations of common gastrointestinal diseases.
